# Governance function analysis of the Patriotic Health Movement in China

**DOI:** 10.1186/s41256-019-0126-y

**Published:** 2019-11-18

**Authors:** Xuan Zhao, Beibei Yuan, Yahang Yu, Weiyan Jian

**Affiliations:** 1 China Center for Health Development Studies, Peking University, Beijing, China; 2 School of Public Health, Peking University, Beijing, China

**Keywords:** Health system governance, Patriotic health movement, Health policy, Political will, China

## Abstract

**Background:**

Health system governance is critical to the operation of a country’s health system and its overall performance. This study analyzes the role of health system governance in driving health policy innovation and effective implementation.

**Methods:**

A retrospective review is applied to collect, analyze and synthesize information from publications and policy documents relevant to the implementation of a typical health policy, the Patriotic Health Movement.

**Results:**

The analysis of governance highlighted a number of features underpinning this policy. These included highest authority prioritizing health system development, specific health policies being prioritized within the national development agenda, strong political will to promote the policies drawing on the advantages of the highly hierarchal administrative system in China, and accumulating evidence from local experience to support policy making**.** It was also found that the formation of these governance practices and how they drove policy innovation and implementation were both closely related to the political and socio-economic contexts in China.

**Conclusion:**

Given that many low- and middle- income countries are strengthening their health systems aimed at UHC, this study demonstrates that along with drawing lessons from health policies or interventions, addressing factors in each governance domain is critical in adapting the policy design to other settings and the effective operation of policies in other settings.

## Background

There is increasing evidence that health system governance is critical to the operation of health systems and their overall performance [1, 2]. Experience from a range of countries suggests that governance has acted as a driver of success in countries that have achieved major advances in health and access to care compared to others with a similar level of wealth [3, 4]. Most health system frameworks attribute a central role to governance [5], as a key function, although there is a considerable lack of clarity as to what it entails compared with other building blocks of the health system.

There have been increasing number of conceptualizations of health system governance [2, 6, 7]. WHO defines governance as both a cross-cutting function of the health system, and one of the six key functions (blocks) of the health system common to all health systems. The concepts of leadership and governance are thus inextricably linked: “governance involves ensuring strategic policy frameworks exist and are combined with effective oversight, coalition building, regulation, attention to system design and accountability.” [8] This implies that the government is seen as the key actor committing to and overseeing advances in health and managing relationships with other actors involved in the health policy formulation and implementation. Under an effectively governed health system, agreed policy goals are more likely to be translated into policies and activities that bring benefits to the majority of the population including excluded groups. In addition, policies are more likely to be well designed, and governments are more able to plan, manage, regulate and implement them [8–10].

There has been increasing efforts to operationalize and assess good governance—often going beyond the health system. Islam’s [11] approach has two components: the national governance framework relevant to all sectors, including voice and accountability, political stability etc., and health specific dimensions, including information and assessment capacities, policy formulation and planning. Another framework for assessing good governance was developed by Siddiqi and covered 10 dimensions [12]. WHO also developed indicators measuring health system governance in 2010, which were rule-based (governance determinants, e.g. existence of essential medicines list) and outcome-based (governance outcomes, e.g. availability of essential medicines in health facilities). Mikkelsen-Lopez [5] adopted WHO’s framework on health systems, and developed another framework to assess the governance in health systems. This combined the building blocks of health systems with five governance elements (participation and consensus, orientation strategic vision and system, addressing corruption, transparency, accountability) [13].

Despite the considerable variability in the different conceptualizations of governance, there is also a considerable overlap between them. For example, accountability, information or knowledge, participation or collaboration were nearly all covered by the frameworks, but there were differences as to whether they were seen as a precondition to governance, a central feature or outcome of its operation. This reflects a tension between the understanding of governance as a framework that underpins all health system functions, and as a separate block in its own right but intersecting with other blocks – with specific interventions within the governance building block of the health system influencing positively other blocks. Furthermore, it is unclear as to what extent health systems governance is shaped by broader processes in society including the rule of law and population participation.

Since the founding of the People’s Republic of China (P.R.C) in 1949, China has made great but uneven progress in strengthening the health system in order to promote Universal Health Coverage (UHC). In order to strengthen the public health system, the Patriotic Health Movement (PHM) started in the 1950s, which successfully mobilized everyone to improve environmental sanitation and to change health behaviors. This effectively controlled deadly infectious diseases in a short period [14] by mobilizing both the supply and demand sides. On the provider / supply side, the Patriotic Health Movement Committee was set up to coordinate all providers and administration departments related to health, and all departments worked together to eliminate diseases [15]. On the demand side, all residents all over the country were mobilized to “improve sanitation”, including cleaning up garbage, drinking clean water, and the appropriate disposal of human waste. These efforts directly contributed to the control of epidemics, such as encephalomyelitis, malaria, measles and typhoid, in rural areas from the middle and late 1960s to the late 1970s [15]. Afterwards, the Campaign was transformed from a mass movement to institutionalized routine work.

Although there are several studies that describe the concrete content of this policy, the underlying factors behind the policies have not been explored. The role of governance in designing and implementing such a wide-scale policy has received less attention.

With the focus on understanding the governance mechanisms that have underpinned these policies rather than their specific design, potential lessons from China can be highlighted and explored in order to transfer them to other countries [16]. The PHM is an especially good case to understand the health system governance in China, since in 2017, the WHO presented the Chinese Government with the Outstanding Model Award for Health Governance in recognition of the achievements of this campaign [17].

This paper explains how the health system governance contributed to the innovation and effective inception, implementation and scaling up of the PHM policy across China, and provides implications for other low- and middle-income countries (LMICs) aimed at strengthening their health systems in the pursuit of UHC.

## Methods

This study is a retrospective analytical review of the development of the PHM in China aimed at understanding the governance practices that led to the design, planning and implementation of this policy.

### Conceptual framework

The study was also guided by the WHO’s framework on health system governance and leadership [8, 16] (Table 2), but also reflecting closely on the seminal work on governance by Siddiqi et al. [12] Variants of this framework have been widely used to conceptualize the functions and key actions of governments and other key actors in relation to each domain, aimed at strengthening the health system. The six key domains of governance were further broken down into specific sub-questions, concepts and search terms by a multi-disciplinary international expert group aimed at synthesizing the experience of China in health system development and lessons for other LMICs. Close attention was paid to finding matching terms for each domain so that they are clear and meaningful in relation to the local policy framework and published research. The development of the conceptual framework was carried out through an initial face-to-face workshop followed by a virtual interaction to refine the framework.

### Search sources

We searched four electronic databases (PubMed (1966 to 14 December 2018), Proquest Dissertations & Theses Database (1861 to 14 December 2018), China National Knowledge Infrastructure (CHKD-CNKI, 1915 to 14 December 2018) and Chinese Medicine Premier (Wanfang Data, 1988 to 14 December 2018) for literature published in English or Chinese without publication date restrictions. We also searched the websites of the China Health and Family Planning Committee, the WHO, and the World Bank. The search strategy and terms in English and Chinese are listed in Table 1. Historical policy documents were obtained from the archives of the China Health and Family Planning Committee and other related ministries. We also consulted experts in the field of health system strengthening and rural health care in China to identify additional relevant materials and policy documents.

**Table 1:** Search strategy

Databases searched	Search terms
PubMed (Search date: 14 December 2018)	(((politics [MH] OR Organization and Administration [MH] OR Decision Making [MH] OR Consensus [MH] OR Ethics [MH] OR Lobbying [MH] OR governance [TIAB] OR “policy making”[TIAB] OR “policy-making”[TIAB] OR “policy maker”[TIAB] OR “policy makers”[TIAB] OR “policy-maker”[TIAB] OR “policy-makers”[TIAB] OR decision-maker [TIAB] OR “decision making”[TIAB] OR “decision makings”[TIAB] OR decision-makers [TIAB] OR decentralization [TIAB] OR decentralisation [TIAB] OR decentralized [TIAB] OR decentralised [TIAB] OR recentralization [TIAB] OR recentralisation [TIAB] OR centralization [TIAB] OR centralisation [TIAB] OR administrator [TIAB] OR administrators [TIAB] OR government [TIAB] OR governments [TIAB] OR regulation [TIAB] OR regulations [TIAB] OR stakeholder [TIAB] OR stakeholders [TIAB] OR responsiveness [TIAB] OR accountability [TIAB] OR equity [TIAB] OR inequity [TIAB] OR inequities [TIAB] OR leadership [TIAB])) AND (CMS [TIAB] OR NCMS [TIAB] OR NRCMS [TIAB] OR “Cooperative Medical System”[TIAB] OR “New Rural Cooperative Medical System”[TIAB] OR “health insurance”[TIAB] OR “health system reform” [TIAB] OR “healthcare reform” [TIAB] OR “Patriotic Health Movement”[TIAB] OR (health [TIAB] AND reform [TIAB]))) AND (China [MH] OR China [TIAB]) 78 new items
Proquest (Search date: 14 December 2018)	((ti (politics OR governance OR “policy making” OR “policy-making” OR “policy maker” OR “policy makers” OR “policy-maker” OR “policy-makers” OR decisionmaker OR “decision making” OR “decision makings” OR decisionmakers OR decentralization OR decentralisation OR decentralized OR decentralised OR recentralization OR recentralisation OR centralization OR centralisation OR administrator OR administrators OR government OR governments OR regulation OR regulations OR stakeholder OR stakeholders OR responsiveness OR accountability OR equity OR inequity OR inequities OR leadership) AND (ti (CMS OR NCMS OR NRCMS OR “Cooperative Medical System” OR “New Rural Cooperative Medical System” OR “health insurance” OR “health system reform” OR “healthcare reform” OR “Patriotic Health Movement”) OR ti (health AND reform))) OR (ab (politics OR governance OR “policy making” OR “policy-making” OR “policy maker” OR “policy makers” OR “policy-maker” OR “policy-makers” OR decisionmaker OR “decision making” OR “decision makings” OR decisionmakers OR decentralization OR decentralisation OR decentralized OR decentralised OR recentralization OR recentralisation OR centralization OR centralisation OR administrator OR administrators OR government OR governments OR regulation OR regulations OR stakeholder OR stakeholders OR responsiveness OR accountability OR equity OR inequity OR inequities OR leadership) AND (ab (CMS OR NCMS OR NRCMS OR “Cooperative Medical System” OR “New Rural Cooperative Medical System” OR “health insurance” OR “health system reform” OR “healthcare reform” OR “Patriotic Health Movement”) OR ab (health AND reform)))) AND (ti (China) OR ab (China) OR diskw (China) OR su (China) OR au (China) OR sch (China)) 3 new items
China National Knowledge Infrastructure (CHKD-CNKI) (Search date: 14 December 2018)	(主题 = 治理 + 管理 + 分权 + 集权 + 政府 + 规定 + 规则 + 规划 + 利益相关者+((设计 + 执行 + 制定) NEAR (方案 + 政策 + 制度))) AND (主题 = 医疗 + 健康 + 卫生 + 合作医疗+新农合 + 新医改)
Chinese Medicine Premier (Wanfang Data) (Search date: 14 December 2018)	(题名:(“治理” + “管理” + “设计” + “执行” + “制定” + “分权” + “方案” + “政策” + “政治” + “政府” + “规则” + “利益相关者”))*(题名:(“合作医疗” + “新农合”+” 新型农村合作医疗” + “爱国卫生” + “新医改” + “医疗卫生体制改革” + “卫生体系” + “卫生系统”))

### Inclusion criteria

Our aim was to include all studies analyzing the governance practices of PHM, however very few studies exist that focus primarily on the governance aspects of PHM. In order to obtain sufficient information for the study objectives, we included all articles describing or analyzing the initiation, design and scaling up the PHM, and then extracted information related to governance characteristics. Deciding on the relevance of papers on health system governance was based on whether they could provide information on the WHO’s definition of governance and its functions (Table 2) [8, 16]. In this WHO framework, the leadership and governance of health systems involve six key functions common to all health systems: policy guidance, system design, regulation, intelligence and oversight, accountability and collaboration (Table 2).

**Table 2:** Health system governance functions and the specific attributes of each domain [8, 12, 18]

Governance domain	Content of each domain	Specific questions guiding the review and data analysis
Policy guidance and vision	Formulating sector strategies and also specific technical policies; defining goals, directions and spending priorities across services; identifying the roles of public, private and voluntary actors and the role of civil society.	Any long term health plans /development goals/ any documents? Role of “Health” in overall country development? Any other technical plans or strategies for this policy? Are goals and priorities clearly defined in the policy strategies? Are the policy strategies and designs comprehensive? Who initiated the policy (e.g. central government, departments and level, individual, or media)? Why initiated the policy (e.g. problems targeted, or high level will)?
System design	Ensuring a fit between strategy and structure and reducing duplication and fragmentation.	Is the specific health policy related to overall health or national development plan? To what extent does the system structure respond to what needs to happen in this policy? Design of interventions and policies to deliver services Are the duplication and fragmentation reduced?
Regulation and management capacity	Designing regulations and incentives and ensuring they are fairly enforced.	How is the policy operationalized (e.g. regulation, contract or legal)? How can the different levels of government accountable to the policy aims (e.g. supervision, promotion of leader) be ensured? How is the administrative structure organized and how does it contribute to the enforcement of policy (e.g. duties allocation, reporting to higher authority) What regulations or incentives ensure its implementation? How can the management capacity of different levels of governments be ensured (e.g. selection, training, supervision)
Accountable and transparent	Ensuring all health system actors are held publicly accountable. Transparency is required to achieve real accountability.	How can the health policy be responsive to current health priorities? How can the different levels of governments adopt policies based on the needs of local residents? Is the performance of health authorities or health providers transparent to the public? Are the financial situation of health authorities or health providers or health insurance funds transparent to the public?
Intelligence and oversight	Ensuring generation, analysis and use of intelligence on trends and differentials in inputs, service access, coverage, safety; on responsiveness, financial protection and health outcomes; on the effects of policies and reforms; on the political environment and opportunities for action; and on policy options.	What is the basis for policy design and content? Does evidence underpin policy design? How are pilots evaluated? How are the local experience and lessons formulated and disseminated? What monitoring and evaluation systems are in place? How do the monitoring and evaluation contribute to policy?
Collaboration and coalition building	Across sectors in government and with actors outside government, including civil society, to influence action on key determinants of health and access to health services; to generate support for public policies, and to keep the different parts connected - so called ‘joined up government.	How do the actors work across different sectors on design and implementation of policy? How are different actors mobilized to contribute to health activities? What is the level of collaboration with civil society organizations or other (outside the formal health sector)? How are the different levels of government collaborating?

There were no restrictions on the study designs and methods included, and we did not conduct a quality appraisal including a risk of bias, as our aim was not to quantify any research results and assess the impact of governance. Our appraisal of the quality of primary documents was in terms of the significance and relevant information [18].

**Fig. 1:**
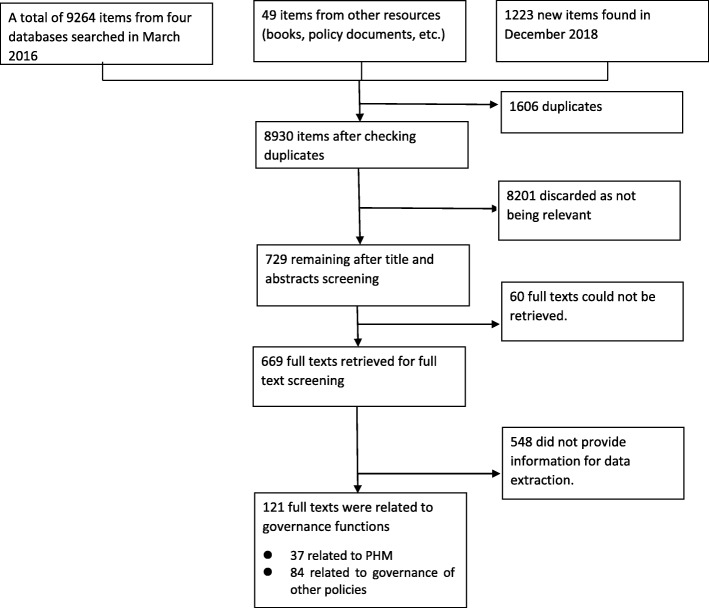
The literature and document selection process

Two assistants independently screened the abstracts and titles, and discussed the findings with the lead author in order to achieve consensus. The lead author screened all the full texts and other co-authors checked all the full texts in order to ensure that no important documents had been missed. A total of 9313 studies were retrieved from the first search conducted on March 3, 2016. We also updated the search on 14 December 2018 and found 1223 new items. Based on the above inclusion criteria, we kept 729 potentially relevant papers after screening these 10,536 titles and abstracts. We then examined the full texts of the potential relevant literature. Finally, 37 closely relevant papers on PHM were included for data extraction and analysis. The selection process is shown in Fig. 1.

### Data extraction and synthesis

For each domain of the conceptual framework and for each question related to these domains, the governance practices or factors closely shaping the design and implementation of the policy were extracted and described. We analyzed and synthesized the information extracted using a framework synthesis approach [19, 20]. The rationale of this method is that for the large amounts of textual data extracted from primary studies, a framework synthesis offers a highly structured approach to organizing and analyzing data. Framework synthesis needs an a priori framework, which is used to extract and synthesize findings, and the new attributes or dimensions can be developed from primary materials, which are incorporated into the previous framework. In our synthesis process, firstly, we categorized the governance strategies related to PHM into corresponding attributes listed in the framework (Table 2). New attributes were added if they were not included in the original framework. The tables were then used to summarize all the governance strategies applied during the PHM process (Table 3).

**Table 3:** Governance practices underpinning two specific health policies

Governance domain	Specific criteria in each domain	PHM
Policy guidance and vision [21, 22, 24, 28–32]	Long term health plans /development goals/strategies Role of this health policy in overall country development Policy initiated from populations’ problems Technical plans or strategies for this policy Goals and priorities clearly defined in the policy strategies Policy strategies and designs define the roles of different actors	There was clear health sector development strategy, but no formal long-term health sector development plan. It was regarded as a crucial policy for national security and development. Initiated by government based on need for national defense and due to the serious infectious disease situation. Several official documents were issued by central government to direct local governments to design and implement CMS. Control infectious disease and defeat the germ war were the final aims of this policy. The policy design guidance documents included requirements on the participating actors and departments, but their specific roles were not detailed.
System design [23, 33, 34]	Specific health policy is related to overall health or national development plan Health system structure responds to what needs to happen in this policy Duplication and fragmentation are avoided and reduced?	Related to national health development strategy Forming a new administration system for this policy in order to ensure the implementation. The new administration department was organized by members from other relevant departments.
Regulation and management capacity [35–49]	Appropriate system or measures to ensure the policy to be operationalized (e.g. regulation, contract, legal or incentives) System or measures to ensure the different levels of government accountable to the policy aims System or measures to ensure the management capacity of different levels of governments	Issuing regulations and rules for top-down implementation. Top-down way: Supervision, strict inspection from higher authority. No documents provided relevant information.
Accountable and transparent [41, 47, 50–53]	System or measures to ensure different levels of governments to adopt policies based on the needs of local residents? The performance of health authorities or health providers is transparent to the public The finance situation of health authorities or health providers or health insurance fund is transparent to the public	Local authorities were encouraged adopted the PHM design based on local situation. In some areas, the performance of relevant institutions was ranked and reported to public. No documents provided relevant information.
Intelligence and oversight [37, 49, 54–57]	Evidence basis is available for policy design and contents Monitoring and evaluation systems are in place and contribute to policy	No evidences support at the beginning, but in the process of implementation, the policy was improved and refined based on evidences from typical areas. The reporting system for infectious diseases was always built during the implementation of this policy in some areas.
Collaboration and coalition building [29, 58, 59]	Different government departments work together on design and implementation of policy Different social actors outside government contribute to the design and implementation of policy	Different government departments were involved in initiating the PHM and design, under coordination from the highest decision makers. Some unions, such as the labor union, women’s union, also participated in the implementation of the PHM.

After we had synthesized the governance strategies based on the above framework, we then explained and discussed under what contextual environments, which governance strategies contributed the appropriate plan and effective implementation of PHM and how. The analysis and explanation process was iterative and led to modifications in the sub-domains of the framework. When the governance attributes in the framework were not supported by evidence, two senior health system researchers with specialist knowledge in this area, and one policymaker who was involved in design and implementation of PHM, provided expert advice on supplementing the information from additional sources. These experts and policy makers were also consulted to validate the analysis, and to interpret how the governance practices worked under certain contexts. Importantly, the findings were analyzed as a process, to establish plausible associations and to account for time lags between policy developments, outcomes and outputs.

## Findings

Here, we firstly describe the context and development of PHM, and then the specific governance practices underpinning this policy are described and compared.

### Background context to PHM

In 1949 the People’s Republic of China was established. China was economically undeveloped after a long period of war. Because the People’s Republic of China was established and the threat of war still existed, the development of national defenses was China’s priority. Poor health was the biggest challenge facing China in terms of strengthening the fighting capacity of the army. The life expectancy was 35 years old, with the greatest health challenges including a high prevalence of infectious diseases. Maternal mortality was 1500/100,000 and the infant mortality rate was 200/1000. In addition, health was regarded by the country’s leader as important to promote the country’s social and economic development. Consequently, in terms of public policies, public health was given the highest priority [21, 22]. In addition, the health system was poorly developed at that time: health facilities and human resources were scarce and unevenly distributed, mainly concentrated in the urban areas. There were only 1400 county hospitals in over 2200 counties nationwide. In rural areas, the density of hospital beds was 0.05 per 1000 population, with a few private facilities available (0.73 per 1000 population), and there was an acute shortage of medicines. There was less than one doctor (trained in western medicine) per 100,000 people, and these were largely concentrated in major coastal cities and provincial capitals [23–25].

### Content and achievements of PHM

The aim of PHM was to control infectious diseases by improving environments, changing people’s health knowledge, and encouraging healthy behaviors by extensive social mobilization.

The content of this policy changed according to the public health problems in different periods. From 1952 to 1954, the policy was based on eradicating the media pests of infectious diseases, such as the plague, cholera, and typhoid; with compulsory immunization, water source protection; and encouraging the maintenance of a clean environment. From 1955 to 1967 the policy focused on eradicating the intermedium, changing those behaviors and social traditions that were detrimental to health, thereby improving the environment.

The Patriotic Health Movement was stopped during the Culture Revolution and restarted in 1979. The policy content since 1979 has been very comprehensive, covering nearly all public health management issues and health promotion actions, such as improving drinking water and toilets, upgrading the infrastructure and living facilities, environmental protection, health education, infectious disease control, etc.

In 1989, as a response to the WHO Healthy Cities Project, in big cities and regions, the China Health City (CHC) Project was started as an effort to strengthen the Patriotic Health Movement and to improve urban living conditions. The Patriotic Health Movement Committee is responsible for the daily management of the CHC project, and in order to win the CHC award, cities have to meet a list of environmental and health indicators [26].

No studies have used rigorous method to evaluate this comprehensive policy. However it has been broadly accepted that the Patriotic Health Movement has contributed greatly to quickly controlling rampant infectious diseases and effectively lowering morbidity. The statistics show that by the end of 1997, the water supply system had benefited 850 million people, 25.4% of excrement and urine was treated, which was double that of 1992. In the 1940s, schistosomiasis was widespread in 12 provinces, and more than 400 counties. With the efforts of PHM, schistosomiasis was eradicated in 1958 [27]. It was reported that China eradicated smallpox 16 years ahead of other Asian and African countries.

One study evaluating the CHC project found that the CHC initiative was associated with increases in the proportion of treated urban domestic sewage (32%), and the proportion of treated urban domestic garbage (30%) [26]. The health status of the population in China also improved although this was attributed to the comprehensive health system in addition to PHM: the mortality rate was 25 per 1000 in the 1950s which decreased to 6.57 per 1000 in the 1990s; the infant mortality rate decreased from 200 per 1000 to 31.4 per 1000 at the end of the last century; the average life expectancy increased from 35 in 1949 to 70 in 2000 [27].

### Policy guidance and vision

Based on WHO’s framework of health system governance, the government should ensure that its policy aims are clear and of a high priority in terms of the country’s development, and it should also provide explicit guidance on how to plan and design the policy content [8].

The PHM was started in 1952 when China had just entered a period of peace after long-term war and devastation: the infrastructure had been destroyed and economic development was extremely slow. Deadly infectious and parasitic diseases were also very common. As discussed in the majority of studies, the PHM was regarded as a crucial policy for national security and development [21, 22].

Firstly, the explicit aim of this policy is to mitigate the risks of serious infectious disease outbreaks, which had led to a large number of deaths and loss of labor, thus holding back economic development [24]. In addition, under the threat of the germ war (China government predicted the risk of other countries using germs attacking China at that time), the Chinese government regarded epidemic prevention and a clean environment as a strategy to combat this threat, which is commonly accepted among academics as the reason why China launched the PHM [21, 22, 28]. The planning and implementation of many actions highlighted the government’s commitment to this policy. At the national level, China’s Prime Minister took the role of director of the national patriotic health committee, and at the local level, the patriotic health committees were also directed by the top leader of the local government [29]. PHM was also included in several national development plans, including “Twelve Years Health Work Plan” and “National Plan for Agricultural Development” in the 1950s [30, 31]. In addition, in order to identify the specific actions and provide a clear direction for local governments in the implementation of the policy, a series of documents were also issued by central government [23].

“*The highest leader of the Communist Party in each area should directly lead the epidemic prevention campaign, and take actions against the germ war.*” From “The Instructions on How to be a Strong Leader in the Epidemic Prevention Campaign” issued by Jiangxi Province Communist Party Committee [32].

### System design

In WHO governance framework, health policy with system design should build or adjust its delivery system of health services and organizational structure in order to respond to the implementation and aims of this policy. In addition, when the structure is built or adjusted, duplication and fragmentation should be avoided [8].

In order to implement the PHM, the first step of each government level was to establish the administration departments from the top to bottom levels. In mid-March 1952, the central government established a “central epidemic prevention committee” (changed to “patriotic health committee” in 1953) [23]. At the end of March, local epidemic prevention committees were established in the level of provinces (the largest administrative geographical area in China) and municipality (the second largest). In the following months, each county, township and village (From the largest to smallest, the administrative levels in China are province, municipality, county, township and village in sequence.) all quickly established epidemic prevention administration departments. For example, In 1952, 433 administrative townships in Jinhua City, Zhejiang Province, all established epidemic prevention departments [33].

In order to mobilize all the population, different industries and organizations also established departments to organize this epidemic prevention work [23]. Usually the committees at different levels were comprised of not only a health department, but also all the relevant departments based on the content of the PHM, which highlights how the management structure was designed in accordance with policy aims. For example, in 1952 in Hebei Province, the core PHM work included the prevention of epidemics, quarantine in ports, quarantine of entomophily, and epidemic status reports. Hebei province thus set up a committee, which included agriculture, health, education, transportation, and public security departments [34].

### Regulation and management capacity

This governance function ensures the enforcement of the health policy, including the regulations and incentives used to guide the behaviors of different levels of government and the various actors involved [8].

The administrative system of China was very hierarchical at that time, and thus the highest administrative level usually forwarded the directives, regulations or work requirements to lower administrative levels [35, 36]. After the PHM directive in 1953 [37] issued by the State Council, the first step was to establish an administrative and implementation system from the top down. The effectiveness of top-down directions or policy documents was stronger if the policy was endorsed by the highest level. For the PHM, the strong push from the highest leader, Mao Zedong, was an important driving force. Before the issue of the State Council’s directions, Mao Zedong even personally advocated “*mobilizing all resources and populations, to pay attention to sanitation, to reduce the prevalence of infectious diseases and improve the health status, and to defeat the germ war*”. (Literature Research Office of the CPC Central Committee 1989) [38].

Under this hierarchical administrative system, supervision and inspections from the top down, as a form of administration pressure, was the most common measure to ensure the enforcement of policies [39, 40]. For example, between April to September of 1952, Nanjing City established over 1000 inspection groups and conducted over 12,000 inspections, covering 2400 companies and 200,000 households. After inspections from the highest government level, the appraisals and ranking were usually based on performance: those performing well would be rewarded financially and recommended as representative successful cases [41–43].

Another kind of incentive to mobilize all the citizens and institutions to implement the PHM was to inspire their patriotic emotions. At first, the government simply explained the health situation, but without obtaining the desired results [14, 44–46]. From the beginning of 1952 the focus turned to inspiring patriotism and anti-war feeling by introducing the threat of a germ war and the importance of PHM in combating this threat [47]. The incentive power of patriotism was effective since China had just experienced a long war, and because the population’s anti-war feeling was strong and this had inspired a high level of patriotism.

*“All citizens are required to participate in epidemic prevention work, including cleaning work, eliminating flies, mosquitoes, delousing and fleas etc.; and the epidemic prevention work should be more emphasized in cities and vital transportation areas.”* From a Directive issued by the State Council of the Central Government and People's Revolutionary Military Committee [48].

*“Every committee at the village or township level should organize appraisals once a year and reward the well-performing organizations/families/individuals once a year, and provincial committees should organize appraisals and reward the well-performing cities/counties every two years.”* From the Handbook for the Patriotic Health Movement issued by Hunan Province Patriotic Health Movement [49].

### Accountability and transparency

This governance function aims to ensure that all the relevant actors are held publicly accountable.

In the PHM, several procedures were designed to ensure that the local government would be accountable for how effectively the PHM was implemented. Firstly, frequent inspections from higher administration levels were combined with critical reports of those that performed poorly [50]. In addition, in some areas the performance of different districts or institutions were ranked and reported to the public by different kinds of media [41, 51]. As one documented case shows, during one inspection of the food industry’s sanitation conditions in Shenyang City in April of 1952, some stores with poor sanitation were broadcast to the public [47, 52].

“*This measure applies to all health institutions, factories, mining sites, schools, troops, state-owned businesses, the food industry; all individuals etc. … The institutions who did not conduct the required work were warned... People were fined 0.5 yuan for spitting, and those leaving cigarettes burning in the street were fined 0.5 yuan … ”* (From a report on the reward and punishment measures of the Beijing Patriotic Health Movement) [53].

### Intelligence and oversight

A supportive governance system defined by WHO framework uses intelligence and evidence in policy generation, implementation and impact evaluation [8].

In the 1950s, the academic research and intelligence resources in China were not very well developed, and the design of PHM was conducted by central government based on limited experience of epidemic prevention. Central government thus just established the policy direction and various principals regarding the policy content [54]. For example the total content of the government’s “Instructions on carrying out the Patriotic Health Movement in 1953” [37] consisted of fewer than 1000 words. This document did not propose detailed operational plans or guidelines, but just emphasized that every province should put forward a concrete local plan of the PHM before January 1953. A review of various provincial policy plans on local patriotic health work revealed more specific goals, tasks and supporting measures [49, 55], however documentation on how the local governments used the evidence or other intelligent sources to design the specific policy is not available [56].

In view of the importance of monitoring in relation to epidemic prevention, policymakers started to build data collection and reporting systems for epidemic diseases. For example in Hebei Province, the data collection platform was built by epidemic prevention committees the first of which was set up in 1952. Telephones and telegrams were used to report epidemic information and had to be reported at the provincial level within 24 h for a area on the plains, and 48 h for a mountainous area. The policy documents issued after 1955 reveal that a three-tier health delivery system was in operation, which started to take the responsibility for collecting information on epidemics. However, there are few documents showing how the information system really worked [57].

### Collaboration building

This governance function requires cooperation between the government sectors and external actors, jointly support the generation and implementation of public policies [8].

The PHM was a typical policy involving the cooperation between different sectors in China’s health system. The PHM work involved many sectors, such as health, the environment, and food and agriculture [58]. From its start, the highest level decision makers realized that without the cooperation of multiple sectors, the aims of PHM would never be achieved. When patriotic health committees started to be established at every level, central government required that they be led by the highest leader in local government, and be comprised of each relevant government department director along with representatives of trade unions and other youth and women’s groups [29]. For example in Shanghai, the mayor acted as the director of Shanghai’s patriotic health committee; associate mayors acted as associate directors; and department leaders of public security, civil administration, public work, financing, culture, education, health, federations of trade unions, women’s unions, students’ unions, and the association of industry and commerce also participated as committee members [59].

“*In 1957, Shanxi Province issued “Notification about Implementing the Sudden Eradication Campaign against the Four Pests (rats, flies, mosquitoes, and sparrows) in Spring”, all the relevant departments, including the department of agriculture, health department, food department, communist youth league of the provincial Party committee, provincial trade union, and the provincial women’s union, worked together to implement the eradication activities.”*[58]

## Discussion

This study aims to identify how governance structures and mechanisms facilitated the design and implementation of PHM, which was implemented on a large scale and was credited with strengthening the public health system in China. This approach seeks to shift the debate from ‘which’ specific policies worked (which designs, resources, capacities etc.) to ‘what governance mechanisms were in operation’ to foster innovation. Given that many LMICs are striving to strengthen their health systems and the importance of health systems governance in achieving this goal, understanding the multiple ways in which governance can strengthen the health systems, can help to draw transferable lessons to other LMICs seeking to accelerate progress toward UHC.

### How health system governance contributed to policy innovation and implementation

The PHM was initiated in the 1950s, when health system development was considered a priority in China’s development agenda. In the 1950s, in a tense international relationship, the newly-established country badly needed nation defense security and economic recovery, and the poor health of the population was the biggest barrier to these goals. The highest authority, the Communist Party of China (CPC) paid great attention to social issues especially for the large majority of rural residents, and to social equality.

The vision of the highest authority regarding health system is always the most important foundation for health system strengthening in a country like China, where public policies are driven more by the authority’s powerful discretion and less by opinion polls. Only by the highest authority prioritizing health, could the central government offer substantial commitment to the development of health policies, including a strong political and financial commitment. This then became the basis for different departments designing and effectively implementing policies that were more appropriate to local situations.

The specific tools applied by central government to push policy planning and implementation consisted of issuing regulations and assigning policy-relevant tasks to lower government levels. The Directive on the implementation of the Patriotic Health Movement in 1953 detailed the requirements of the policy implementation from the highest to the lower levels of government. Usually the key launching documents were jointly issued by the State Council (the central government) and the CPC Central Committee (the ruling party) with the requirements on the policy implementation from the highest level authority and central government to the lower levels. This was the generic process of any policy scale-up in China where the political system is hierarchical [60]. The effects of these practices on facilitating policy implementation were dependent on the strong commitment of central government and the range of concrete and visible steps undertaken to ensure this commitment n. For example, the directives issued by Mao Zedong played a key role in scaling up this policy, especially the requirement that the highest leader of local government should also be director of the local patriotic health committee; and the inspections from higher level governments resulted in rewards or punishments for local governments based on their performance. It could be argued that effectiveness of any regulation or incentive enforcing the health policies and pursuing faster implementation was reflected by the central government’s commitment and prioritization of the policies even where they do not have an implementation capacity.

Another specific governance practice to facilitate the scaling up of policies consisted of the establishment of a special administration system. China covers a vast territory with varied development levels from province to province. Even if the central government has a strong political will, it is therefore very difficult to push forward a top-down policy within a short time. In order to guarantee the execution of PHM, China has established a specialized administrative system: the Patriotic Health Movement Committee Office. The tasks of the provincial, municipal, district and county governments to promote the local PHM were thus undertaken and driven by these administration departments. The government budget provides the salaries for the personnel in these systems. These specific administration systems are embedded in a hierarchical system to ensure that the central government’s decision can be quickly passed to a local department, and grassroots implementation information can also be quickly sent up.

The combination of central government’s top design and local autonomy in terms of the specific design is key in promoting the policy innovation. Given that China is a country with vast territory and regional differences, it is impossible to apply a one size fits all policy design. Such a combination of constraints regarding the top framework and local authorities influenced local governments in two channels: the higher level governments regularly supervised and monitored the implementation status of the lower governments to ensure they complied with the central government’s top design. At the same time the higher-level governments established the areas which worked well as a benchmark to encourage other areas. The benchmarking model mobilized the local governments to explore measures adapted to local conditions, and to take initiative in the implementation of the central government policy [61].

Finding the intelligence and information sources to support policy design is another important function of health system governance. During the whole process of health system strengthening, one typical practice was to accumulate evidence from local practices implemented in different parts of the country [61]. The well-performing pilot schemes resulted in recognition and promotion by central government. Another reason for this type of governance practice was that in the 1950s, China was not open in terms of international relationships, and China had little interest in learning from other countries. In addition, with limited resources and supportive institutions, the applicability of experiences from other countries was also low. Thus the approach was to encourage different areas to pilot actions and to summarize the evidence from those explorations. This governance practice led to policy innovation and effective implementation in three ways: by facilitating the mobilization of intellectual resources at all levels in the design of the initial policy; the recognition of local policy variations may have encouraged local governments to pursue the most appropriate policy design; and thirdly, the policy design was continuously refined based on practices within the country that were more acceptable to different sectors and to different contexts, therefore accelerating and facilitating implementation.

Our analysis of the level of collaboration generated a number of good practices associated with the rolling out of the PHM in China. The PHM achieved substantial department collaboration in the policy planning and implementation, including jointly issuing the launch directives. In addition the establishment of the management committee was composed of directors from all relevant departments, and citizens and resources were mobilized from every sector to implement the related policy actions. It could be argued that without the prioritization of health policies and coordination from central government, department collaboration would not have been possible.

Despite China’s hierarchical top-to-down health system, national governance is still fragmented, with decision-making distributed in different governmental departments who have powers in relation to key functions, including technical support and supervision, financial support decisions, and personnel management. In this system, there are different political pressures among various interest group and departments, which have to compete for political and economic resources [62]. This kind of adversarial national governance is not conducive to reaching consensus and coordinating efforts among departments towards a common goal. Some health system reforms involving many departments are thus still slow to make progress in China today. Department collaboration should be able to provide the potential breakthrough for the reform of China’s health system governance.

### Comparisons with framework we applied and other studies

This paper has applied a governance framework to identify the specific governance practices which supported the effective implementation of the PHM. The information extraction followed a rigorous search strategy and relied on a transparent screening process. We synthesized description information on the design and implementation process of the PHM based on six domains of health system governance, with the concept of governance translated into specific questions. In the process of matching data and themes to the various governance questions, classifying the governance practices related to PHM formulation and implementation, to the specific governance functions was critical because the description information did not involve any governance analysis.

One problem that arose during this process was that the same practices can be classified under different governance functions. For example, local government autonomy in specific policy design can be seen as an expression of local accountability to local needs. On the other hand it is also relevant for “intelligence source” because this practice can mobilize more evidence resources for policy design. These discrepancies were discussed within the team, and in collaboration with senior researchers, and the final classification was revised through regular contact with the experts with experience of governance analysis in other settings. The findings of our review were also validated by senior researchers and policy makers who were involved in the implementation of PHM.

Another issue was that for some governance domains, we found no description information on the PHM that showed the corresponding governance features, such as “measures to ensure the management capacity”.

Several emerging themes were not relevant to any of the specific questions we originally devised, for example, “patriotic emotion” applied to mobilize citizens people was not directly relevant to any question. After a discussion within the team and with experts, we put “patriotic emotion” under “regulation” because “regulation” was broadly defined as any “regulations” or “incentives” ensuring that policies were fairly enforced.

Studies on the policy design of PMH and its impacts on health system performance in China [26, 63], have often reported that cooperation between departments along with mass mobilization were key to the success of the PHM. However, no studies have used governance concepts to analyze these practices during the design and implementation of the PMH.

The governance perspective has been used in other LMICs [64–66] to analyze health system policies. In these studies, decentralization, citizen participation and a broader stakeholder base, clear focus of policy design, and the capacity of policy makers were all concluded as key determinants for the implementation of health policies.

In our study, the key governance practices in China we found included a strong commitment by national institutions through regulations and policy targets aimed at sub-national government authorities, together with cooperation between departments. The differences between China and other LMICs can be attributed to different political backgrounds and administration structures.

### The implications for other low- and middle-income countries

Most LMICs, which have a different economic and social development status compared to China, are trying to strengthen their health systems. The experience of China in implementing the PHM provides a range of useful lessons on how to design innovative policies based on local contexts and on how to implement the policies effectively. Firstly, at the design stage, encouraging sub-national governments to pilot policy options and accumulate evidence and report experiences from local practices, and to compare them with different administrative areas could help to test and identify a range of policy options which fit with local health systems, and are feasible and acceptable to different actors. Secondly, clear accountability and relationships can help to synthesize sub-national experiences and ensure it shapes national strategies. Thirdly, at the stage of scaling up schemes nationally, leadership by national institutions through regulations and policy goals and targets should be aimed at sub-national government authorities, using performance in policy implementation as the evaluation criteria of local government officers, and encouraging local government to pilot innovative measures can offer leverage. A caveat is that this strategy may be more applicable in more hierarchical administrative systems, although it should be noted that China combines centralized decision making with considerable autonomy at the province level. Fourthly, an administration system specially designed for certain policies may promote a rapid scale-up but duplication and the waste of resources should be prevented. Finally, the most important point is that central government should explicitly prioritize the health system and specific health policies in an overall national development agenda, which is the basis for the effective planning and implementation of policies.

## Conclusion

This study sought to identify how governance structures and mechanisms have enabled the design and implementation of the Patriotic Health Movement on a large scale and credited with a major contribution to strengthening the public health system in China. A number of governance features underpinning this policy were found, including highest authority prioritizing health system development, specific health policies being also prioritized within national development agenda, strong political to promote the policies drawing on the advantages of the highly hierarchal administrative system in China, and accumulating evidences generated from local experience to support policy making. It was also found that the mechanism of how these practices working on pushing the policy innovation and implementation was closely related to political and socio-economic development contexts in China. Given that many LMICs are strengthening their health systems to achieve the goals of UHC, this study demonstrates that compared to drawing lessons from the contents of health policies or interventions, addressing factors in each governance domain is critical for the adaptation of the policy design to other settings and for the effective operation of policies in other settings.

## Data Availability

All the data and materials involved in this paper are from the published articles, and they are all available online.
